# Specific function and modulation of teleost monocytes/macrophages: polarization and phagocytosis

**DOI:** 10.24272/j.issn.2095-8137.2019.035

**Published:** 2019-05-18

**Authors:** Xin-Jiang Lu, Jiong Chen

**Affiliations:** 1 1 Laboratory of Biochemistry and Molecular Biology, School of Marine Sciences, Ningbo University, Ningbo Zhejiang 315211, China; 2 2 Key Laboratory of Applied Marine Biotechnology of Ministry of Education, Ningbo University, Ningbo Zhejiang 315211, China

**Keywords:** Teleost, Monocytes /Macrophages, Phagocytosis, Cytokine production, Comparative immunology

## Abstract

Macrophages exist in most tissues and play a variety of functions in vertebrates. Teleost fish species are found in most aquatic environments throughout the world and are quite diverse for a group of vertebrate animals. Due to whole genome duplication and environmental adaptation, teleost monocytes/macrophages possess a variety of different functions and modulations compared with those of mammals. A deeper understanding of teleost monocytes/macrophages in the immune system will not only help develop teleost-specific methods of disease prevention but will also help improve our understanding of the various immune mechanisms in mammals. In this review, we summarize the differences in polarization and phagocytosis of teleost and mammalian macrophages to improve our understanding of the various immune mechanisms in vertebrates.

## INTRODUCTION

Macrophages exist in most animal tissues, in which they play a variety of functions. They are known by different names in various groups, such as amebocytes, hemocytes, coelomocytes, granulocytes, monocytes, and macrophages, but have similar morphology and comparable functions (Bilej et al., 2000; Wiegertjes et al., 2016). Macrophages are best known for their role in immunity, as elucidated by Eli Metchnikoff in the late nineteenth century (Tauber, 2003). Several studies have highlighted the wide range of functions of macrophages in vertebrate biology, including systemic metabolism, cold adaptation, tissue homeostasis, and development (Okabe & Medzhitov, 2016; Wynn et al., 2013). The basic functions of macrophages in vertebrates are cytokine production and phagocytosis (Hodgkinson et al., 2015). In both mammals and fish, monocytes give rise to macrophages during inflammatory conditions (Hodgkinson et al., 2015). Moreover, macrophage colony stimulating factor (CSF1) plays a crucial role in macrophage growth and differentiation in both mammals and fish (Hodgkinson et al., 2015). Teleosts are among the evolutionarily oldest vertebrates, possessing both innate and classical adaptive immune systems (Dickerson & Findly, 2017). Environmental factors have been shown to affect the immune system of teleosts (Makrinos & Bowden, 2016), which are widespread in most aquatic environments. Various immune genes in non-model teleosts have been identified, with transcriptome and genome development (Mackintosh & Ferrier, 2017; Qian et al., 2014; Shao et al., 2016), providing an opportunity to conduct studies on the adaptive evolution of the immune system. In this review, we focus on the differences in polarization and phagocytosis of teleost and mammalian macrophages, which should help in the development of a new perspective on macrophages and their role in adaptive evolution.

### DEFINITION OF MONOCYTES/MACROPHAGES IN TELEOSTS

Monocytes/macrophages in mammals are an important component of the mononuclear phagocytic system, and play diverse roles during infection, inflammation, and tissue injury and repair (Okabe & Medzhitov, 2016). In mammals, protein markers are used to identify monocytes, macrophages, and dendritic cells by flow cytometry. Monocytes, which mainly exist in bone marrow, blood, and spleen, can further differentiate into inflammatory macrophages and dendritic cells during inflammation (Ray & Rai, 2017; Shi & Pamer, 2011). Their migration to tissues and differentiation into inflammatory macrophages and dendritic cells are determined by the inflammatory milieu and pattern recognition receptors on the cells (Murray, 2018). Macrophages reside in a variety of tissues, including lymphoid and non-lymphoid tissues. They are equipped with a broad range of pattern recognition receptors to facilitate cytokine production and phagocytosis during inflammatory responses (Franz & Kagan, 2017). Classical dendritic cells are specialized antigen processing and presenting cells that exhibit high phagocytic activity as immature cells and high cytokine-producing capacity as mature cells (Qian & Cao, 2018).

In teleosts, monocytes/macrophages have been identified in a variety of species, including rainbow trout (*Oncorhynchus mykiss*) (Leal et al., 2017), zebrafish (*Danio rerio*) (Yu et al., 2017), goldfish (*Carassius auratus* L.) (Hodgkinson et al., 2017), and ayu (*Plecoglossus altivelis*) (Lu et al., 2017). The human CD14 antigen is highly expressed in monocytes and to a lesser extent in macrophages (Shi & Pamer, 2011; Zhang et al., 2017). However, CD14 has not been identified in the teleost genome (Novoa et al., 2009). Hence, it is difficult to discriminate between teleost monocytes and macrophages based on protein markers using flow cytometry. Stafford et al. (2001) developed a method using cell-conditioned medium to obtain monocyte-like cells in rainbow trout. However, it is unclear whether monocytes can be identified from the culture of teleost monocytes/macrophages based on acute isolation using simple procedures, such as flow cytometry. Although dendritic-like cells have been identified in rainbow trout (Soleto et al., 2018), it is unclear whether such cells exist in other dominant teleost species. As monocytes and macrophages share similar characteristics, most teleost investigations have defined the adhered mononuclear cells of the kidney or head kidney as monocytes/macrophages or macrophages.

Phagocytosis is the defining characteristic of macrophages (large eaters), as classified by Metchnikoff (Tauber, 2003). However, most phagocytes among the peripheral blood mononuclear cells of teleosts are IgM^+^ B lymphocytes, not monocytes/macrophages (Li et al., 2006). Thus, morphological analysis, apart from phagocytosis index analysis, is necessary to identify the monocytes/macrophages in teleosts.

## MACROPHAGE POLARIZATION AND CYTOKINE PRODUCTION

In vertebrates, the inflammatory response of macrophages plays an important role against pathogens (Geissmann et al., 2010; Ricci et al., 2018). Macrophage polarization against different inflammatory stimuli depends on environmental cues or pathophysiological conditions (Lawrence & Natoli, 2011). Classically activated macrophages (M1 type) are induced by lipopolysaccharides (LPSs), a major component of the outer membrane of gram-negative bacteria, and IFN-γ, and express pro-inflammatory mediators. Conversely, alternatively activated macrophages (M2 type) are induced by IL-4 and IL-13, and express high levels of anti-inflammatory mediators (Shapouri-Moghaddam et al., 2018). Macrophage polarization is also regulated by soluble proteins, intracellular signals, and transcription factors. Galectin-dependent regulatory signaling stimulates M2-type macrophage polarization (Blidner et al., 2015). Toll-like receptor (TLR) signaling activates the signal transducer and activator of transcription (STAT) 1 protein to skew macrophage function towards the M1 type, whereas activation of STAT3 by IL-4 and IL-13 skews macrophage function towards the M2 type (Sica & Mantovani, 2012). Similarly, the ablation of protein kinase B α (PKBα/Akt1) or protein kinase B β (PKBβ/Akt2) differentially affect macrophage polarization (Arranz et al., 2012). Tissue milieus with M1 type are biased towards cell-mediated cytotoxicity, whereas the term “M2 type” is used for a variety of conditions that inhibit M1 type (Yamaguchi et al., 2015). The immune milieus are skewed to M2 type in some tissues, like the gills in teleosts and uterus in pregnant mammals (Yamaguchi et al., 2015).

Monocyte/macrophage polarization has also been detected in teleosts (Wiegertjes et al., 2016). Cytokines participate in teleost monocyte/macrophage polarization, particularly IFN-γ and IL-4/IL-13 (Wiegertjes et al., 2016). Moreover, inducible nitric oxide synthase (iNOS) is a marker for M1 type and arginase 2 is a marker for M2 type in teleost monocytes/macrophages (Wiegertjes et al., 2016). In carp, the expression of pro-inflammatory cytokines IL-1β, TNF-α, and CXCa, peak in peritoneal leukocytes 24 h after zymosan induction, whereas the expression of anti-inflammatory mediators IL-10 and arginase 2 peak 96 h and 168 h after zymosan induction, respectively (Chadzinska et al., 2008). This suggests that monocytes/macrophages display both classic- and alternative pathway-induced polarization upon immune stimulation *in vivo*. Monocyte/macrophage polarization in teleosts has also been investigated *in vitro*. LPSs from gram-negative bacteria are probably the best studied microbial stimuli for macrophage activation. However, the mammalian LPS receptor, TLR4, may not be functional in teleosts (Sepulcre et al., 2009). The presence of LPSs may be sensed by other mechanisms in teleosts, as LPSs are still thought to be important immune stimulators (Meng et al., 2012). Nitrite production in *in vitro* monocyte/macrophage culture is up-regulated after LPS stimulation in teleosts (Joerink et al., 2006). Other pro-inflammatory mediators, such as IL-1β, IL-12, and iNOS are also up-regulated in LPS-stimulated teleost monocytes/macrophages (Joerink et al., 2006), suggesting that LPS induces an M1-type polarization in teleost monocytes/macrophages. In mammals, M2-type polarization is mainly induced by IL-4, IL-13, parasite infection, CSF1, TGF-β, and IL-10 (Sica & Mantovani, 2012). Apart from anti-inflammatory mediators, cAMP plays an important role in M2-type monocyte/macrophage polarization signaling (Bystrom et al., 2008). In teleosts, at least two IL-4/IL-13 genes exist (IL-4/13A and IL-4/13B), both with low homology to IL-4 and IL-13 (Ohtani et al., 2008). In goldfish, recombinant IL-4/13 has been found to induce arginase activity and down-regulate the nitric oxide (NO) response in primary monocytes/macrophages (Hodgkinson et al., 2017), suggesting IL-4/13 functions to induce M2-type polarization in teleost monocytes/macrophages. In teleost carp (Joerink et al., 2006) and ayu (Chen et al., 2018), cAMP has also been employed to successfully induce M2-type monocyte/macrophage polarization.

Several new mechanisms for macrophage polarization have been identified recently. Mammals possess single IFN-γ molecules that are important in the activation of M1-type macrophages (Grayfer et al., 2018). Many teleosts have multiple IFN-γ molecules, some of which are elicitors of reactive oxygen species (ROS) but not NO, whereas others elicit NO but limited ROS (Grayfer et al., 2018). Although functional analogs of the mammalian M1/M2 macrophage subsets are present in teleosts, defining the regulatory mechanisms governing the polarization of these effector populations is a far more challenging goal (Grayfer et al., 2018). Many immune genes exist in two copies in the teleost genome due to genome duplication (Aghaallaei et al., 2010). These redundant genes may result in sub-functionalization, as in the case of European sea bass (*Dicentrarchus labrax*), in which different hepcidins exhibit different roles (Neves et al., 2015). We observed that ayu has two CXCR3 genes, which contribute to monocyte/macrophage polarization (Lu et al., 2017). In mammals, the chemokine receptor CXCR3 exists as a single gene, and is preferentially expressed on immune cells to aid in cell migration to the sites of inflammation (Bromley et al., 2008). Furthermore, CXCR3.1 (CXCR3b) and CXCR3.2 (CXCR3a) are found in zebrafish (*Danio rerio*), Japanese ricefish (*Oryzias latipes*), spotted green pufferfish (*Tetraodon nigroviridis*), ayu (*Plecoglossus altivelis*), and grass carp (*Ctenopharyngodon idella*) (Aghaallaei et al., 2010; Lu et al., 2017). However, more research is necessary to illustrate the teleost-specific mechanisms of monocyte/macrophage polarization.

### PHAGOCYTOSIS BY MONOCYTES/MACROPHAGES

Phagocytosis is an important cellular process for the induction of antimicrobial responses and regulation of adaptive immunity (Rieger et al., 2012). After phagocytosis, both teleost and mammalian macrophages show pro-inflammatory and homeostatic responses (Rieger et al., 2012). Mammalian neutrophils have the capacity to internalize apoptotic bodies, whereas teleost neutrophils do not possess the same activity (Rieger et al., 2012). Most studies have shown that in fish, monocytes, macrophages, and neutrophils are the main phagocytic cells, as found in mammals (Esteban et al., 2015). Furthermore, phagocytosis in teleosts has been observed in several other kinds of cells, including B-1 cells (Li et al., 2006), γδ-T cells (Wan et al., 2016), and thrombocytes (Nagasawa et al., 2014). Although mammalian phagocytosis has also been observed in B-1 cells (Parra et al., 2012) and γδ-T cells (Wu et al., 2009), its presence in thrombocytes seems unique to teleosts. It is well established that teleost monocytes/macrophages generate reactive oxygen intermediates as part of antimicrobial defense, similar to that observed in mammalian macrophages (Hodgkinson et al., 2015). However, significant differences between goldfish and mice have been observed in response to pro-inflammatory and homeostatic internalization signals during phagocytosis of immune cells (Rieger et al., 2012). When European sea bass (*Dicentrarchus labrax*) monocytes/macrophages have been incubated with different pathogenic agents, different pathogens have been observed to have different effects on monocyte/macrophage activity (Bennani et al., 1995). However, further investigation is necessary to illustrate the specific mechanisms of pathogen recognition and phagocytosis in teleosts.

Phagocytosis in mammals is triggered by pathogen recognition, which is a complex process involving a variety of pattern-recognition receptors. The main pattern-recognition receptors include lectin-like recognition molecules, C-type lectins, scavenger receptors, mannose receptors, CD14, and Toll-like receptors (Uribe-Querol & Rosales, 2017). In teleosts, the pathogen recognition mechanism is different from that of mammals. LPSs are sensed by a variety of pattern-recognition receptors (Ranf, 2016) present on the cell surface (Triantafilou & Triantafilou, 2002). Phagocytosis in macrophages is regulated by LPS recognition receptors, such as TLR4 (Lv et al., 2017) and CD14 (Lingnau et al., 2007). TLR4 was the first receptor identified to be involved in the recognition of LPSs. TLR4 must be associated with myeloid differentiation protein 2 (MD2) for interaction with LPSs, and activation of TLR4 is preceded by the transfer of LPSs to CD14 by an LPS-binding protein (Neyen & Lemaitre, 2016). However, CD14 and MD2 do not exist in teleost genomes (Iliev et al., 2005). Thus, it remains unclear how teleost monocytes/macrophages compensate for the CD14 function during phagocytosis of pathogens.

## FUTURE DEVELOPMENTS

**Figure 1:**
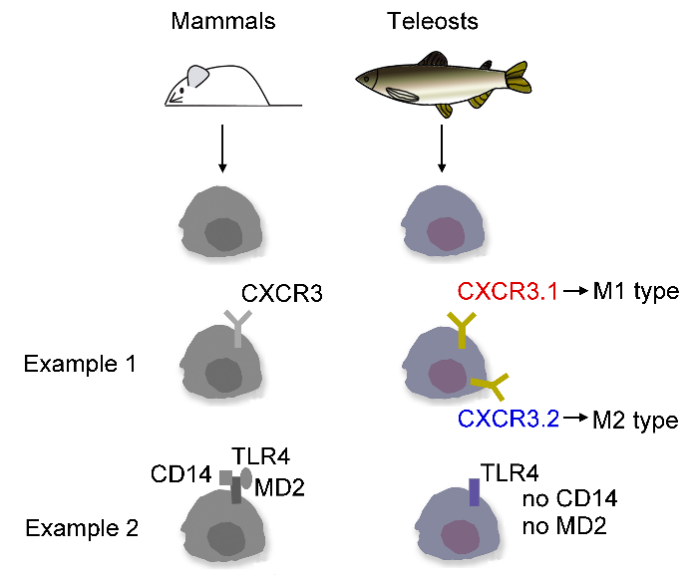
Differences in monocytes/macrophages between mammals and teleosts

Teleost fish species are found throughout the world and are quite diverse for a group of vertebrate animals. Monocytes/macrophages are the basic immune cells not only in mammals but also in teleosts. Monocytes/macrophages are easy to isolate, purify, and segregate, providing us with a useful tool for understanding the differences between the immune systems of mammals and teleosts. Although the basic function of monocytes/macrophages is similar in vertebrates, there are a variety of different points between mammals and teleosts (Figure 1). These differences have likely arisen from genetic factors, such as whole genome duplication, and environmental adaptations. Whole genome duplication in teleosts produces a variety of redundant genes, which may be sub-functional during adaptive evolution. Gene function in teleosts is modulated by environmental factors, such as salinity, hypoxic conditions, and temperature. Therefore, future investigations regarding the mechanisms of teleost monocyte/macrophage polarization and phagocytosis under the influence of various environmental factors are necessary. A deeper understanding of the teleost immune system will not only help develop teleost-specific methods of disease prevention but will also help improve our understanding of the various immune mechanisms within mammals.

## References

[B1-ZoolRes-40-3-146] Aghaallaei N, Bajoghli B, Schwarz H, Schorpp M, Boehm T 2010 Characterization of mononuclear phagocytic cells in medaka fish transgenic for a cxcr3a: gfp reporter. Proceedings of the National Academy of Sciences of the United States of America, 107(42): 18079–18084. 20921403 10.1073/pnas.1000467107 PMC2964234

[B2-ZoolRes-40-3-146] Arranz A, Doxaki C, Vergadi E, Martinez De La Torre Y, Vaporidi K, Lagoudaki ED, Ieronymaki E, Androulidaki A, Venihaki M, Margioris AN, Stathopoulos EN, Tsichlis PN, Tsatsanis C 2012 Akt1 and Akt2 protein kinases differentially contribute to macrophage polarization. Proceedings of the National Academy of Sciences of the United States of America, 109(24): 9517–9522. 22647600 10.1073/pnas.1119038109 PMC3386059

[B3-ZoolRes-40-3-146] Bennani N, Schmid-Alliana A, Lafaurie M 1995 Evaluation of phagocytic activity in a teleost fish, *Dicentrarchus labrax* . Fish & Shellfish Immunology, 5(3): 237–246.

[B4-ZoolRes-40-3-146] Bilej M, De Baetselier P, Beschin A 2000 Antimicrobial defense of the earthworm. Folia Microbiologica, 45(4): 283. 11347248 10.1007/BF02817549

[B5-ZoolRes-40-3-146] Blidner AG, Méndez-Huergo SP, Cagnoni AJ, Rabinovich GA 2015 Re-wiring regulatory cell networks in immunity by galectin-glycan interactions. Febs Letters, 589(22): 3407–3418. 26352298 10.1016/j.febslet.2015.08.037

[B6-ZoolRes-40-3-146] Bromley SK, Mempel TR, Luster AD 2008 Orchestrating the orchestrators: chemokines in control of T cell traffic. Nature Immunology, 9(9): 970–980. 18711434 10.1038/ni.f.213

[B7-ZoolRes-40-3-146] Bystrom J, Evans I, Newson J, Stables M, Toor I, Van Rooijen N, Crawford M, Colville-Nash P, Farrow S, Gilroy DW 2008 Resolution-phase macrophages possess a unique inflammatory phenotype that is controlled by cAMP. Blood, 112(10): 4117–4127. 18779392 10.1182/blood-2007-12-129767 PMC2581990

[B8-ZoolRes-40-3-146] Chadzinska M, Leon-Kloosterziel KM, Plytycz B, Lidy Verburg-Van Kemenade BM 2008 *In vivo* kinetics of cytokine expression during peritonitis in carp: evidence for innate and alternative macrophage polarization. Developmental & Comparative Immunology, 32(5): 509–518. 17988736 10.1016/j.dci.2007.08.008

[B9-ZoolRes-40-3-146] Chen F, Lu XJ, Nie L, Ning YJ, Chen J 2018 Molecular characterization of a CC motif chemokine 19-like gene in ayu (*Plecoglossus altivelis*) and its role in leukocyte trafficking. Fish & Shellfish Immunology, 72: 301–308. 29128493 10.1016/j.fsi.2017.11.012

[B10-ZoolRes-40-3-146] Dickerson HW, Findly RC 2017 Vertebrate adaptive immunity—comparative insights from a teleost model. Frontiers in Immunology, 8: 1379. 29123524 10.3389/fimmu.2017.01379 PMC5662878

[B11-ZoolRes-40-3-146] Esteban MÁ, Cuesta A, Chaves-Pozo E, Meseguer J 2015 Phagocytosis in teleosts. implications of the new cells involved. Biology, 4(4): 907–922. 26690236 10.3390/biology4040907 PMC4690022

[B12-ZoolRes-40-3-146] Franz KM, Kagan JC 2017 Innate immune receptors as competitive determinants of cell fate. Molecular Cell, 66(6): 750–760. 28622520 10.1016/j.molcel.2017.05.009 PMC5503483

[B13-ZoolRes-40-3-146] Geissmann F, Manz MG, Jung S, Sieweke MH, Merad M, Ley K 2010 Development of monocytes, macrophages, and dendritic cells. Science, 327(5966): 656–661. 20133564 10.1126/science.1178331 PMC2887389

[B14-ZoolRes-40-3-146] Grayfer L, Kerimoglu B, Yaparla A, Hodgkinson JW, Xie J, Belosevic M 2018 Mechanisms of fish macrophage antimicrobial immunity. Frontiers in Immunology, 9: 1105. 29892285 10.3389/fimmu.2018.01105 PMC5985312

[B15-ZoolRes-40-3-146] Hodgkinson JW, Fibke C, Belosevic M 2017 Recombinant IL-4/13A and IL-4/13B induce arginase activity and down-regulate nitric oxide response of primary goldfish (*Carassius auratus* L.) macrophages. Developmental & Comparative Immunology, 67: 377–384. 27581741 10.1016/j.dci.2016.08.014

[B16-ZoolRes-40-3-146] Hodgkinson JW, Grayfer L, Belosevic M 2015 Biology of bony fish macrophages. Biology, 4(4): 881–906. 26633534 10.3390/biology4040881 PMC4690021

[B17-ZoolRes-40-3-146] Iliev DB, Roach JC, Mackenzie S, Planas JV, Goetz FW 2005 Endotoxin recognition: in fish or not in fish?. Febs Letters, 579(29): 6519–6528. 16297386 10.1016/j.febslet.2005.10.061 PMC1365396

[B18-ZoolRes-40-3-146] Joerink M, Ribeiro CMS, Stet RJM, Hermsen T, Savelkoul HFJ, Wiegertjes GF 2006 Head kidney-derived macrophages of common carp (*Cyprinus carpio* L.) show plasticity and functional polarization upon differential stimulation. The Journal of Immunology, 177(1): 61–69. 16785499 10.4049/jimmunol.177.1.61

[B19-ZoolRes-40-3-146] Lawrence T, Natoli G 2011 Transcriptional regulation of macrophage polarization: enabling diversity with identity. Nature Reviews Immunology, 11(11): 750–761. 10.1038/nri3088 22025054

[B20-ZoolRes-40-3-146] Leal E, Zarza C, Tafalla C 2017 Effect of vitamin C on innate immune responses of rainbow trout (*Oncorhynchus mykiss*) leukocytes. Fish & Shellfish Immunology, 67: 179–188. 28602736 10.1016/j.fsi.2017.06.021

[B21-ZoolRes-40-3-146] Li J, Barreda DR, Zhang YA, Boshra H, Gelman AE, Lapatra S, Tort L, Sunyer JO 2006 B lymphocytes from early vertebrates have potent phagocytic and microbicidal abilities. Nature Immunology, 7(10): 1116–1124. 16980980 10.1038/ni1389

[B22-ZoolRes-40-3-146] Lingnau M, Höflich C, Volk HD, Sabat R, Döcke WD 2007 Interleukin-10 enhances the CD14-dependent phagocytosis of bacteria and apoptotic cells by human monocytes. Human Immunology, 68(9): 730–738. 17869646 10.1016/j.humimm.2007.06.004

[B23-ZoolRes-40-3-146] Lu XJ, Chen Q, Rong YJ, Chen F, Chen J 2017 CXCR3.1 and CXCR 3 2 differentially contribute to macrophage polarization in teleost fish. The Journal of Immunology, 198(12): 4692–4706. 10.4049/jimmunol.1700101 28500070

[B24-ZoolRes-40-3-146] Lv JZ, He XY, Wang HT, Wang ZH, Kelly GT, Wang XJ, Chen Y, Wang T, Qian ZQ 2017 TLR4-NOX2 axis regulates the phagocytosis and killing of mycobacterium tuberculosis by macrophages. BMC Pulmonary Medicine, 17(1): 194. 29233104 10.1186/s12890-017-0517-0 PMC5727946

[B25-ZoolRes-40-3-146] Mackintosh C, Ferrier DEK . 2017 Recent advances in understanding the roles of whole genome duplications in evolution. F1000 Research, 6: 1623. 10.12688/f1000research.11792.1 PMC5590085 28928963

[B26-ZoolRes-40-3-146] Makrinos DL, Bowden TJ 2016 Natural environmental impacts on teleost immune function. Fish & Shellfish Immunology, 53: 50–57. 26973022 10.1016/j.fsi.2016.03.008

[B27-ZoolRes-40-3-146] Meng Z, Zhang XY, Guo J, Xiang LX, Shao JZ 2012 Scavenger receptor in fish is a lipopolysaccharide recognition molecule involved in negative regulation of NF-κB activation by competing with TNF receptor-associated factor 2 recruitment into the TNF-α signaling pathway. The Journal of Immunology, 189(8): 4024–4039. 22988031 10.4049/jimmunol.1201244

[B28-ZoolRes-40-3-146] Murray PJ 2018 Immune regulation by monocytes. Seminars in Immunology, 35: 12–18. 29290545 10.1016/j.smim.2017.12.005

[B29-ZoolRes-40-3-146] Nagasawa T, Nakayasu C, Rieger AM, Barreda DR, Somamoto T, Nakao M 2014 Phagocytosis by thrombocytes is a conserved innate immune mechanism in lower vertebrates. Frontiers in Immunology, 5: 445. 25278940 10.3389/fimmu.2014.00445 PMC4165319

[B30-ZoolRes-40-3-146] Neves JV, Caldas C, Vieira I, Ramos MF, Rodrigues PNS . 2015 Multiple hepcidins in a teleost fish, *Dicentrarchus labrax*: different hepcidins for different roles. The Journal of Immunology, 195(6): 2696–2709. 26268656 10.4049/jimmunol.1501153

[B31-ZoolRes-40-3-146] Neyen C, Lemaitre B 2016 Sensing gram-negative bacteria: a phylogenetic perspective. Current Opinion in Immunology, 38: 8–17. 26569344 10.1016/j.coi.2015.10.007

[B32-ZoolRes-40-3-146] Novoa B, Bowman TV, Zon L, Figueras A 2009 LPS response and tolerance in the zebrafish (*Danio rerio*). Fish & Shellfish Immunology, 26(2): 326–331. 19110060 10.1016/j.fsi.2008.12.004 PMC2748242

[B33-ZoolRes-40-3-146] Ohtani M, Hayashi N, Hashimoto K, Nakanishi T, Dijkstra JM 2008 Comprehensive clarification of two paralogous interleukin 4/13 loci in teleost fish. Immunogenetics, 60(7): 383–397. 18560827 10.1007/s00251-008-0299-x

[B34-ZoolRes-40-3-146] Okabe Y, Medzhitov R 2016 Tissue biology perspective on macrophages. Nature Immunology, 17(1): 9–17. 26681457 10.1038/ni.3320

[B35-ZoolRes-40-3-146] Parra D, Rieger AM, Li J, Zhang YA, Randall LM, Hunter CA, Barreda DR, Sunyer JO 2012 Pivotal advance: peritoneal cavity B-1 B cells have phagocytic and microbicidal capacities and present phagocytosed antigen to CD4^+^ T cells. Journal of Leukocyte Biology, 91(4): 525–536. 22058420 10.1189/jlb.0711372 PMC3317272

[B36-ZoolRes-40-3-146] Qian C, Cao XT 2018 Dendritic cells in the regulation of immunity and inflammation. Seminars in Immunology, 35: 3–11. 29242034 10.1016/j.smim.2017.12.002

[B37-ZoolRes-40-3-146] Qian X, Ba Y, Zhuang QF, Zhong GF 2014 RNA-Seq technology and its application in fish transcriptomics. Omics A Journal of Integrative Biology, 18(2): 98–110. 24380445 10.1089/omi.2013.0110 PMC3920896

[B38-ZoolRes-40-3-146] Ranf S 2016 Immune sensing of lipopolysaccharide in plants and animals: same but different. PLoS Pathogens, 12(6): e1005596. 27281177 10.1371/journal.ppat.1005596 PMC4900518

[B39-ZoolRes-40-3-146] Ray R, Rai V 2017 Lysophosphatidic acid converts monocytes into macrophages in both mice and humans. Blood, 129(9): 1177–1183. 28069607 10.1182/blood-2016-10-743757

[B40-ZoolRes-40-3-146] Ricci C, Ruscica M, Camera M, Rossetti L, Macchi C, Colciago A, Zanotti I, Lupo MG, Adorni MP, Cicero AFG, Fogacci F, Corsini A, Ferri N 2018 PCSK9 induces a pro-inflammatory response in macrophages. Scientific Reports, 8(1): 2267. 29396513 10.1038/s41598-018-20425-x PMC5797178

[B41-ZoolRes-40-3-146] Rieger AM, Konowalchuk JD, Grayfer L, Katzenback BA, Havixbeck JJ, Kiemele MD, Belosevic M, Barreda DR 2012 Fish and mammalian phagocytes differentially regulate pro-inflammatory and homeostatic responses in vivo. PLoS One, 7(10): e47070. 23110059 10.1371/journal.pone.0047070 PMC3479104

[B42-ZoolRes-40-3-146] Sepulcre MP, Alcaraz-Pérez F, López-Muñoz A, Roca FJ, Meseguer J, Cayuela ML, Mulero V 2009 Evolution of lipopolysaccharide (LPS) recognition and signaling: fish TLR4 does not recognize LPS and negatively regulates NF-κB activation. The Journal of Immunology, 182(4): 1836–1845. 19201835 10.4049/jimmunol.0801755

[B43-ZoolRes-40-3-146] Shao CW, Bao BL, Xie ZY, Chen XY, Li B, Jia XD, Yao QL, Ortí G, Li WH, Li XH, Hamre K, Xu J, Wang L, Chen FY, Tian YS, Schreiber AM, Wang N, Wei F, Zhang JL, Dong ZD, Gao L, Gai JW, Sakamoto T, Mo SD, Chen WJ, Shi Q, Li H, Xiu YJ, Li YZ, Xu WT, Shi ZY, Zhang GJ, Power DM, Wang QY, Schartl M, Chen SL 2016 The genome and transcriptome of Japanese flounder provide insights into flatfish asymmetry. Nature Genetics, 49(1): 119–124. 27918537 10.1038/ng.3732

[B44-ZoolRes-40-3-146] Shapouri-Moghaddam A, Mohammadian S, Vazini H, Taghadosi M, S-A Esmaeili, Mardani F, Seifi B, Mohammadi A, Afshari JT, Sahebkar A 2018 Macrophage plasticity, polarization, and function in health and disease. Journal of Cellular Physiology Banner, 233(9): 6425–6440. 10.1002/jcp.26429 PMC13482560 29319160

[B45-ZoolRes-40-3-146] Shi C, Pamer EG 2011 Monocyte recruitment during infection and inflammation. Nature Reviews Immunology, 11(11): 762–774. 10.1038/nri3070 PMC3947780 21984070

[B46-ZoolRes-40-3-146] Sica A, Mantovani A 2012 Macrophage plasticity and polarization: *in vivo* veritas. Journal of Clinical Investigation, 122(3): 787–795. 22378047 10.1172/JCI59643 PMC3287223

[B47-ZoolRes-40-3-146] Soleto I, Fischer U, Tafalla C, Granja AG 2018 Identification of a potential common ancestor for mammalian cross-presenting dendritic cells in teleost respiratory surfaces. Frontiers in Immunology, 9:59. 29422901 10.3389/fimmu.2018.00059 PMC5788898

[B48-ZoolRes-40-3-146] Stafford JL, Mclauchlan PE, Secombes CJ, Ellis AE, Belosevic M 2001 Generation of primary monocyte-like cultures from rainbow trout head kidney leukocytes. Developmental & Comparative Immunology, 25(5–6): 447–459. 11356224 10.1016/s0145-305x(01)00015-5

[B49-ZoolRes-40-3-146] Tauber AI 2003 Metchnikoff and the phagocytosis theory. Nature Reviews Molecular Cell Biology, 4(11): 897–901. 10.1038/nrm1244 14625539

[B50-ZoolRes-40-3-146] Triantafilou M, Triantafilou K 2002 Lipopolysaccharide recognition: CD14, TLRs and the LPS-activation cluster. Trends in Immunology, 23(6): 301–304. 12072369 10.1016/s1471-4906(02)02233-0

[B51-ZoolRes-40-3-146] Uribe-Querol E, Rosales C 2017 Control of phagocytosis by microbial pathogens. Frontiers in Immunology, 8:1368. 29114249 10.3389/fimmu.2017.01368 PMC5660709

[B52-ZoolRes-40-3-146] Wan F, Hu CB, Ma JX, Gao K, Xiang LX, Shao JZ 2016 Characterization of γδ T cells from zebrafish provides insights into their important role in adaptive humoral immunity. Frontiers in Immunology, 7(3): 675. 28119690 10.3389/fimmu.2016.00675 PMC5220103

[B53-ZoolRes-40-3-146] Wiegertjes GF, Wentzel AS, Spaink HP, Elks PM, Fink IR 2016 Polarization of immune responses in fish: the ‘macrophages first’ point of view. Molecular Immunology, 69(3): 146–156. 26471699 10.1016/j.molimm.2015.09.026

[B54-ZoolRes-40-3-146] Wu Y, Wu WT, Wong WM, Ward E, Thrasher AJ, Goldblatt D, Osman M, Digard P, Canaday DH, Gustafsson K 2009 Human γδ T cells: a lymphoid lineage cell capable of professional phagocytosis. The Journal of Immunology, 183(9): 5622–5629. 19843947 10.4049/jimmunol.0901772

[B55-ZoolRes-40-3-146] Wynn TA, Chawla A, Pollard JW 2013 Macrophage biology in development, homeostasis and disease. Nature, 496(7446): 445–455. 23619691 10.1038/nature12034 PMC3725458

[B56-ZoolRes-40-3-146] Yamaguchi T, Takizawa F, Fischer U, & Dijkstra JM 2015 Along the axis between type 1 and type 2 immunity; principles conserved in evolution from fish to mammals. Biology, 4(4): 814–859. 26593954 10.3390/biology4040814 PMC4690019

[B57-ZoolRes-40-3-146] Yu T, Guo WL, Tian Y, Xu J, Chen JH, Li L, Wen ZL 2017 Distinct regulatory networks control the development of macrophages of different origins in zebrafish. Blood, 129(4): 509–519. 27940477 10.1182/blood-2016-07-727651

[B58-ZoolRes-40-3-146] Zhang M, Zhu HP, Ding Y, Liu ZY, Cai ZJ, Zou M-H 2017 AMP-activated protein kinase α1 promotes atherogenesis by increasing monocyte-to-macrophage differentiation. Journal of Biological Chemistry, 292(19): 7888–7903. 28330873 10.1074/jbc.M117.779447 PMC5427268

